# Evaluation of Sternal Closure with Absorbable Polydioxanone Sutures in Children

**DOI:** 10.5681/jcvtr.2014.011

**Published:** 2014-03-04

**Authors:** Hamid Bigdelian, Mohsen Sedighi

**Affiliations:** ^1^Department of Cardiac Surgery, Isfahan University of Medical Sciences, Isfahan, Iran; ^2^Department of Cardiovascular Surgery, Shahid Chamran Heart Center, Isfahan University of Medical Sciences, Esfahan, Iran

**Keywords:** Sternal Closure, Polydioxanone, Open Heart Surgery

## Abstract

***Introduction:*** Sternal dehiscence, sternal wound infection and mediastinitis are troublesome complications following median sternotomy which are major causes of morbidity and mortality of patients. Synthetic polydioxanone absorbable suture seems effective in prevention of these complications in children undergoing open heart surgery.

***Methods:*** During 2 years period, 620 patients who underwent median sternotomy were studied. The efficacy of absorbable polydioxanone suture was tested on patients using figure-of-eight suture technique. The patients’ age ranged from newborn to 15 years old. All surgical interventions were performed according to a standard protocol.

***Results:*** No sternal sutures were broken during the sternal closure and no case of mediastinitis was seen. Two patients experienced sternal dehiscence (0.32%). Follow-up period of patients were established between 1 to 132 months after open heart surgery.

***Conclusion:*** Sternal closure with the polydioxanone suture in combination with figure-of-eight technique is a safe and suitable method in children with good clinical results.

## Introduction

Median sternotomy for cardiac surgery was first advocated in 1957 by Julian and colleagues. Since then, it has been the most frequently-used approach in both adult and pediatric patients undergoing open heart surgery.^1^ Sternal dehiscence, superficial wound infection, deep wound infection, and mediastinitis are rare but troublesome complications following median sternotomy approach; they are major causes of morbidity and mortality following open-heart surgery.^2^ Although risk factors for sternal dehiscence include chronic obstructive pulmonary disease (COPD), redo surgery, renal failure, diabetes mellitus, chronic steroid use, obesity, concurrent infection and immunosuppression, intraoperative risk factors such as off-midline sternotomy, prolonged cardiopulmonary bypass, transverse fractures of the sternum have also been identified.^3^

The standard median sternotomy closure involves wiring with stainless steel wires in a figure-of-8 or simple interrupted approach; however, it may not be the ideal approach in patients predisposed to these complications.^4^ Therefore, using absorbable sutures for sternal closure after median sternotomy has developed to a clinically routine procedure. New synthetic polydioxanone absorbable suture (PDS) seems effective in prevention of sternal instability, wound healing and incompatibility in children.^5,6^ The aims of the present study were to evaluate the effect of polydioxanone sutures on wound stability, absence of inflammatory reaction, postoperative pain and suture line bleeding during intensive care unit (ICU) stay, hospital course and follow-up.

## Materials and methods

During 2 years period (2010 to 2012), 620 patients received cardiac surgery via median sternotomy in Chamran Heart Center, Isfahan. Efficacy of an absorbable suture (PDS; Ethicon, Jonson and Jonson, Sommerville, NJ, USA) for sternal closure was tested on patients with figure-of-eight suture technique. Exclusion criteria were redo surgery, additional surgical procedures, reoperation for bleeding, and sternal fractures. The patient ages ranged from newborn to 15 (mean age of 6.57 years old); 350 males and 270 females. The main reason for cardiac surgery via sternotomy was Ventricular septal defect in 267 cases (43%), Atrial septal defect in 99 cases (16%), Tetralogy of Fallot in 85 cases (13.8%), Patent ducts arteriosus in 59 cases (9.5%), coarctation of aorta in 40 cases (6.5%) and other reasons in 70 cases. All the surgical interventions were performed in the same style according to a standard protocol. In order to perform sternal closure, continuous suture technique was used with polydioxanone materials. Study endpoints were the occurrence of sternal dehiscence and wound instability during ICU and hospital stay and follow-up period. The sternal dehiscence diagnosis was made solely on chest radiography showing a mid-sternal strip sign < 3 mm. The main characteristics of the patients are indicates in Table 1.

**Table 1:** Main characteristics of patients

Age	Range	2 Days – 15 years
	Mean ± SD	6.57 ± 8.34 years
	Male	350
Sexuality	Female	270
	Total	620
Weight	Range	2.5 – 45 kg
	Mean ± SD	20.5 ± 2.10 kg

## Results

A total of 620 patients participated in the present study. No sternal sutures were broken during the sternal closure but 2 cases experienced complication and suffered from sternal dehiscence (0.32%). Furthermore, our observations did not show any cases of mediastinitis among the patients during follow up period. Postoperatively, polydioxanone suture caused no inflammatory reaction and mediastinitis, minimal granulation tissue and had a reliable absorption time (range 2-6 months). Follow-up period of whole series of the patients were established between 1 to 132 months in order to evaluate sternal complications ( Table 2).

**Table 2:** Distribution of patients in terms of percentage and sexuality

**Diagnosis**	**No. of patients**	**Percent**	**Male**	**Female**	**Dehiscence**	**Mediastinitis**
Atrial septal defect (ASD)	99	16	38	61	0	-
Ventricular septal defect (VSD)	267	43	187	80	1	-
ASD+VSD	3	0.4	0	3	0	-
Tetralogy of Fallot (TOF)	85	14	36	49	1	-
Transposition of great arteries (TGA)	18	3	8	10	0	-
Pulmonary stenosis (PS)	8	1.3	3	5	0	-
Aortic stenosis (AS)	2	0.3	0	2	0	-
Pulmonary atresia (PA)	14	2.3	9	5	0	-
Total anomalous pulmonary venous connection (TAPVC)	9	1.5	6	3	0	-
Left ventricular dysfunction (LVD)	3	0.4	1	2	0	-
Patent ducts arteriosus (PDA) + VSD	59	9.5	26	33	0	-
Tricuspid atresia (TA)	4	0.6	2	2	0	-
Coarctation of aorta (COA) + VSD	40	6.5	25	15	0	-
Mitral valve replacement (MVR)	1	0.1	1	0	0	-
Aortic valve replacement (AVR)	2	0.3	1	1	0	-
MVR + AVR	1	0.1	1	0	0	-
Pulmonary valve replacement (PVR)	2	0.3	2	0	0	-
Tricuspid valve replacement (TVR)	3	0.4	1	2	0	-
**Total**	**620**	**100**	**347**	**273**	**2**	**0**

## Discussion

Median sternotomy is the approaching method most commonly used in open heart surgery. The sternum is normally closed with non-absorbable sutures such as steel wires with the use of single-passage or figure-of-8 contributing to numerous problems occurring with conventional steel wire passing through the sternum, especially in paediatric cardiac surgery.^7^ Among the complications of median sternotomy, which range from superficial wound infection to mediastinitis, non-infective and mechanical sternal dehiscence are clinically characterized by chest discomfort, sternal splitting and even respiratory distress.^8^ Also, persistent postoperative sternal pain for open heart operation is reported as relatively a common complaint. Risk factors for sternal dehiscence have been classified into three groups. Preoperative risk factors include advanced age and comorbidities, intraoperative factors include prolonged duration of operation and the need for reoperation, and postoperative factors comprise intra-aortic balloon pumping, massive blood transfusion, prolonged mechanical ventilation, and pneumonia while findings indicate that advanced age, obesity, COPD, diabetes mellitus, and renal insufficiency are the main preoperative risk factors.^9,10^

Dehiscence often occurs within the first two weeks postoperatively. However one of the advantages of steel wire is that it is very strong even when a narrow gauge is used and does not need to be knotted. But steel wires may be difficult for the surgeon to handle and may cause pain and discomfort for the children following surgery. Therefore, synthetic absorbable polydioxanone sutures appear effective in terms of handling, strength and lack of inflammatory reactions.^11^ Using absorbable polydioxanone suture in pediatric cardiovascular operation was reported by Myers *et al*. firstly, and now polydioxanone is used in a variety of operations such as aortic coarctation repair, complete repair for anomalous pulmonary veins, transposition of the great arteries and systemic pulmonary shunts. Hence, using absorbable sutures for sternal closure after median sternotomy in children has developed to a clinically routine procedure, because this material has allowed a complication-free stability of the sternum, good wound healing and compatibility.^12-14^ Furthermore, absorbable sutures are easier to handle and reduce the risk of suture lines bleeding and postoperative pain.^13,15,16^ More importantly, this point is noticeable that PDS suture is an efficacious approach in patients with postoperative cardiac arrest that emergency re-sternotomy for internal Cardiopulmonary Resuscitation (CPR) is needed. Although there are different approaches to sternal closure, Figure-of-8 suturing is believed to be more secure and less likely to cut the sternum, because of redistribution of shearing forces compared to simple interrupted closure.^17,18^

In the present study, we evaluated the use of polydioxanone sutures in children. The sternal closure technique was modified to improve the performance and increase the strength retention of the polydioxanone sutures. Altogether, this trial proved that figure-of-eight sternal suturing is equally effective as simple interrupted suturing in preventing sternal dehiscence after open heart surgery. In addition, this absorbable suture is a safe and suitable method for sternal closure in children with good clinical outcome.

## Ethical issues

All patients gave written informed consents and the study was approved by our local Ethics Committee.

## Competing interests

The authors declare no conflicts of interest.
